# The Roles of Motivation and Coping Behaviours in Managing Stress: Qualitative Interview Study of Hong Kong Expatriate Construction Professionals in Mainland China

**DOI:** 10.3390/ijerph15030561

**Published:** 2018-03-20

**Authors:** Isabelle Yee Shan Chan, Mei-yung Leung, Qi Liang

**Affiliations:** 1 Department of Real Estate and Construction, The University of Hong Kong, Pokfulam Road, Hong Kong, China; 2 Department of Architecture and Civil Engineering, City University of Hong Kong, Tat Chee Avenue, Kowloon Tong, Hong Kong, China; bcmei@cityu.edu.hk; 3 Department of Architecture and Civil Engineering, City University of Hong Kong, Tat Chee Avenue, Kowloon Tong, Hong Kong, China; qiliang3-c@my.cityu.edu.hk

**Keywords:** construction, coping behaviours, expatriates, motivations, stress management

## Abstract

Driven by fast-growing economies worldwide, the number of international construction projects is booming, and employing expatriates has inevitably become a strategy used by construction firms. However, stress arising from expatriate assignments can lead to early return, assignment failure, and staff turnover, causing in significant losses to an organisation. Extensive research has focused on the effectiveness of coping behaviours in relation to stress. However, studies investigating the antecedents of coping are rare. The limited studies to date tend to focus on content-based motivations (identifying what), instead of on how coping behaviours can be motivated in the stress management process (identifying how). Focus on expatriate construction professionals (ECPs) is further limited. Hence, this study aims to investigate from a process theory perspective the role of motivation in the stress management process. Using a qualitative interview study approach, involving 22 in-depth interviews, this study first identifies the content of motivation, coping behaviours, performance, and stress in the context of Hong Kong ECPs working on cross-cultural projects in China; it then unveils and explains the associations between the identified variables. Based on the results, stakeholders are recommended to review pre-departure training, so as to ensure that key elements such as personal awareness of stress (cognitive, affective, and physical), expectancies of coping strategies on stress (adaptive or maladaptive), and expectancies of the influence of stress on performance are covered.

## 1. Introduction

Globalisation is not only fostering the interdependence of global economies, but is also increasing the demand for expatriates worldwide. The globalising economy is resulting in more and more international projects, leading to an increased demand for expatriates [1]. This is particularly true for the construction industry, especially in rapidly developing countries like China and India where there are a growing number of global projects demanding high-quality expertise and technology. Employing expatriates has inevitably become a strategy used by many construction firms, particularly those striving to construct innovative buildings to international standards. However, expatriation willingness and performance of employees can be subject to many different factors, such as personal safety, health care, cultural difference and so on [2,3,4]. More importantly, having expatriated staff may not necessarily bring an organisation success. Stress arising from expatriate assignments can lead to problems [5] in terms of assignment failure, early return, and departure from the organisation [6]. It is costly to employ an expatriate; indeed, the cost can be the single largest item of personnel expenditure for a company (save for that spent on the chief executive officer). Therefore, it is important to enhance the performance of expatriate construction professionals (ECPs) by ‘motivating’ them to cope effectively with stress.

Due to the significant changes in work and life brought about by working overseas, expatriates are recognised as a highly-stressed group [7,8]. Such stress can have a significant impact on their performance overseas in terms of poor task performance, poor workgroup relationships, and early return intention [7,8]. Extensive research in the construction and engineering fields has focused on factors affecting expatriation willingness (e.g., [3]), ways to allocate human resources for expatriate assignments (e.g., [9]), and the effectiveness of various coping behaviours of individual professionals (e.g., [10,11]). However, studies of the antecedents of coping behaviours are rare and the limited studies tend to focus on content-based motivations identifying the what—such as work motivation, power motivation, affiliation motivation, and so on—instead of identifying the how—how coping behaviours can be fostered through the stress management process. To ensure high-quality performance, it is important for an expatriate professional to be motivated to cope effectively throughout the stress management process. Hence, this study aims to investigate from a process theory perspective the role of motivation in the stress management process, that is, the coping behaviours-stress-performance process. 

## 2. Performance, Stress, and Coping Efforts

Due to the dynamic nature of construction projects, complicated workgroup relationships and crisis-ridden working environments, the construction industry has long been recognised as a stressful business [12,13,14]. A survey conducted by the Chartered Institute of Building in 2006 revealed that nearly 70% of construction professionals had suffered from stress at work. Expatriate assignments require managers to equip their staff not only with basic task completion strategies, but also the ability to adjust to the changes in work and the difficulties encountered in the unfamiliar host-country environment. As a result, expatriates have been identified as a highly-stressed group [7,8]. However, stress is not necessarily bad. Stress occurs when there is a discrepancy between an individual’s expected and actual ability to deal with his or her job tasks. Though too much stress (expected ability higher than actual ability) can result in “burnout”, too little stress (actual ability higher than expected ability) can also affect an individual through “rustout” [15,16]. Only moderate level of stress can result in optimized level of performance [17]. Therefore, coping, for an individual who suffers from excessive level of stress, may mean stress reduction (distress); while that for an individual who suffers from too little stress, may mean greater eustress. Both burnout and rustout can cause emotional and physical stress symptoms to individuals [18]. 

Stress affects not only an individual’s well-being, but also task and contextual performance in terms of productivity, workgroup cooperation, intention to leave the expatriate assignment before completion (hereafter referred to as early return intention), and so on [19]. To survive in the stressful and challenging construction industry, it is essential for construction professionals and their expatriate managers to make efforts to cope with stress [11,20]. Coping behaviours, or the cognitive and behavioural efforts by an individual to manage stress, are commonly divided into two categories, namely, problem-focused coping behaviour and emotion-focused coping behaviour [21]. 

According to Lazarus and Folkman [21], problem-focused coping refers to an individual’s effort to deal with the sources of their stress, such as planful problem solving, positive reappraisal, and instrumental support seeking; while emotion-focused coping refers to efforts to manage one’s emotional state and maintain moderate levels of arousal, such as escapism, self-control, and emotional support seeking. Researchers have investigated the effectiveness of various problem- and emotion-focused coping behaviours as adopted by construction professionals [11,22] and expatriates [23,24], respectively. In general, problem-focused coping behaviours have been found to relate positively to expatriates’ performance and adjustment, while emotion-focused coping behaviours have negative associations with these outcomes (e.g., [23,24,25]). Although previous research has found that the adoption and effectiveness of a coping behaviour depends on the stressors and environment encountered by an individual [26], motivation has long been recognised as the antecedent factor governing the choice of actions or efforts made by an individual [27,28], not to mention an individual’s efforts to cope.

## 3. Motivation

Motivation has a close association with stress and the performance of individuals in a work context, and includes factors such as stress [29,30], physical health [31], organisational commitment [30], enhanced job satisfaction [32], organisational citizenship behaviours and personal accomplishment [31], entrepreneurial effectiveness [33], and so on. However, the definitions of motivation differ amongst researchers. The term motivation is derived from the Latin movere, which means “to move” [34]. However, as human motivation, cognition, and behaviours are complicated, this single word is not adequate to define the concept of motivation. In the extensive literature on the topic, researchers have defined it in many different ways. For instance, Herzberg [35] defines motivation as the factors causing the satisfaction or dissatisfaction of an individual before he or she makes a decision, while Vrooms [28] describes it as a process governing the choices made by a person towards a voluntary activity.

The variation in definitions can be accounted for by the two approaches to motivation found in the literature, namely, process and content theories. Process theories, like expectancy, equity, and goal-setting theories, focus on the steps and decision processes that individuals go through to put effort into their work (that is, the how) [36,37]. Content theories, including Maslow’s hierarchy of needs, Herzberg’s motivation-hygiene theory, McClelland’s learned-needs theory, and so on, focus on identifying the factors that motivate individuals to put effort into their work (that is, the what) [36,38]. As this study aims to investigate the influence of motivation in the coping behaviours-stress-performance process, a process theory is adopted.

Vroom’s expectancy theory, which focuses on individuals’ conscious and psychological involvement in making choices between various actions [28], is one of the most influential process theories adopted in motivation research [39]. Individuals have expectations and/or anticipations of the outcomes of their behaviours [40] in that they are motivated by the perceived probability of success and the incentive value of such success [41]. Vroom’s expectancy theory was developed based on this proposition. In this theory, expectancy and valence in the motivation process demonstrate the essential cognition of individuals in making choices about their behaviours [28]. Expectancy refers to individuals’ beliefs or expectations about whether a certain course of action or behaviour can result in particular outcomes [42]. The term valence is defined by Vroom [28] as the affective orientations people hold with regard to a particular outcome, which is usually referred to as the importance of a particular outcome [42]. Other researchers have referred to valence as anticipated satisfaction [43,44] and experience satisfaction [45,46].

Hence, according to this theory, if an individual, after assessing the level of effort (E), that he/she needs to put in to result in a certain performance (P) (E → P expectancy), anticipates that performance (P) will lead to certain outcomes (O) (P → O expectancy), and predicts that the outcome is valuable and rewarding (valence), then that individual will be motivated to make an effort to carry out the task.

## 4. The Conceptual Model

According to Lazarus and Cohen’s [47] transactional model of stress and coping, an individual undergoes two levels of appraisal before he or she copes. When encountering a potentially threatening event, he or she first evaluates its significance and judges it in accordance with its positivity, meaningfulness, relevancy, and so on (i.e., primary appraisal). If it is considered to be a stressor, he or she proceeds to evaluate the controllability of the stressor by appraising his or her coping resources or ability (i.e., secondary appraisal). Then, if the individual believes he or she has the resources to cope with the stressor, coping behaviours would be applied. Failure in coping results in stress. The theory denotes the role of coping in mediating the stressor-to-stress relationship [21]. Coping is a result of the stressor-appraisal process, which precedes the manifestation of stress. The experience of stressful situations or stimulus (i.e., stressors) should be differentiated with the manifestation of stress. 

Conforming to the expectancy theory, Lazarus, in the second stage of his transactional model, takes expectancy of coping (effort in E → P expectancy) into account. However, his model does not acknowledge the potential roles of P → O expectancy and valence of outcomes in the stress management process. More importantly, it does not explain how and why different coping behaviours are motivated and adopted in the stress management process. The current study thus aims to further develop the stressor-coping-stress theory through the use of expectancy theory in stress management.

**Figure 1:**
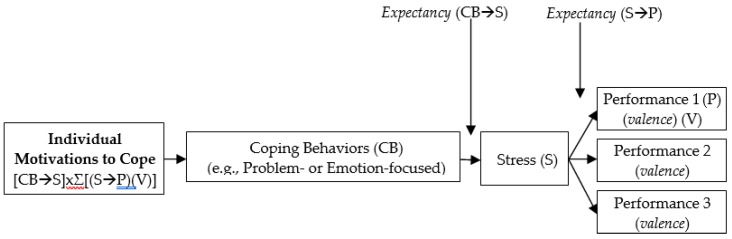
The Conceptual Model for Motivations, Coping Behaviours, Stress and Performance.

Coping behaviours denote the efforts made by an individual to manage stress [21]. The concept is actually congruent with the idea of effort as defined in Vroom’s expectancy theory, which concerns actions taken in response to a broader situation, including stressful situations. Hence, based on this theory, the relationships between individual motivation-to-cope, coping behaviours, stress, and performance can be conceptualised as below (refer to Figure 1). ECPs will be motivated to cope with their stress by undertaking certain coping behaviours (i.e., problem-focused coping behaviors (e.g., planful problem solving, positive reappraisal, and instrumental support seeking) and/or emotion-focused coping behaviors (e.g., escapism, self-control, and emotional support seeking); [21] if they have high expectancy in relation to the effect of that behaviour on their stress (that is, CB → S expectancy), high expectancy in relation to the impact of stress on their performance (S → P expectancy), and high anticipated satisfaction with their performance (valence).

## 5. Research Methods

The qualitative interview study method is suitable for exploring the dynamic relationships between certain causes and effects, with control over the data being sourced from the same project environment [48]. ECPs with different professional roles in selected projects were interviewed individually so as to allow individual variation within the same project environment to be captured, in order to comprehend the effects of the different kinds of tasks for which they were responsible and to allow a comprehensive view of the stress management process they experienced [49]. To further control for the influence of different cultural environments in projects [50], three construction projects with different natures were selected, including: (i) a private project financed by a foreign developer (in Shanghai); (ii) a private project financed by a mainland developer (in Beijing); and (iii) a public project financed by the mainland government (in Guangzhou). 

Driven by its fast-growing economy, the construction industry in mainland China has undergone a boom in recent years. The number of construction firms in mainland China has increased by more than 60% over the last decade [51], leading to a rapid increase in large-scale, innovative construction projects demanding high-quality construction professionals. In addition, the rapid economic development in mainland China has also attracted a large number of foreign investors to invest in international mega projects. Hence, a considerable number of Hong Kong construction professionals have “moved north” to work in these cross-cultural projects as ECPs. 

The term expatriates refers to employees who relocate from their home town to accomplish a job-related goal for their organisation for a specified time period [52]. Although Hong Kong now belongs to mainland China, culturally it lies at a significant distance owing to its 156-year period of colonial rule by the British (from 1841 to 1997). Although the people of Hong Kong have retained some traditional Chinese cultural values, they have also altered or adapted to new cultural values [53]. The cultural distance between the people of Hong Kong and the people of mainland China in terms of collectivism, power distance, uncertainty avoidance, and so on, has been identified [54]. Hence, previous studies refer to Hong Kong Chinese working in mainland China as expatriates who are adjusting to a cross-cultural environment [55,56]. In addition, although the majority of expatriation studies focus on expatriates facing Eastern-Western culture differences, it has been found that assigning a Hong Kong Chinese expatriate to another Chinese culture (though comparatively similar) can be as much, if not more, of a challenging experience as sending them to a Western culture [56]. This is because expatriates that are relocated to a perceived similar culture (but different in actuality) often fail to identify the differences that exist and easily resort to blaming their local colleagues or themselves for problems that are in reality due to cultural conflict [20].

In view of this, three large-scale construction projects in mainland China were selected. The first project was located in Shanghai, which is a representative central business district in mainland China. This large-scale complex project involved the development of a high-standard shopping mall, commercial offices, and a luxury residential area on the site. The project aimed to develop a new landmark in Shanghai. Invested in by a foreign developer, the project attracted a number of foreign construction firms to participate as contractors and/or consultants, resulting in the involvement of a number of ECPs. Eight ECPs taking the roles of developers, contractors, and consultants, were interviewed in this project.

The second construction project was located in Beijing, which is the capital city of mainland China and is also a representative central business district. It was a large-scale, complex project involving the construction of retail shops, offices, and hotels. With the aim of constructing a new landmark for the district, the project not only had an iconic and innovative design, but also achieved a high level of quality and won several awards for energy conservation. Although the main contractor was a local firm, which had not employed any ECPs, a number of expatriate professionals were employed by the developer, sub-contractor, architectural, and consulting firms for this project. Ten interviews were conducted in this project.

The third project was located in Guangzhou, which is the largest city of Guangdong Province in mainland China. The Guangzhou project aimed not only to develop a landmark for local tourism, but also to act as a key part of the city’s infrastructure. This project had a unique architectural design, with its architectural structure breaking the world’s record in a certain aspect. Invested in by the mainland government, the project mainly employed local contractors but involved some input from foreign design and consulting firms. Four ECPs from structural and quantity surveying consulting companies were interviewed.

In order to control the reliability of data collection, purposive sampling was adopted [57]. Interviewees were selected and personally invited based on three criteria: (i) they were not mainland citizens; (ii) they were qualified construction professionals with experience in the construction industry before expatriation; and (iii) they were working in mainland China on these particular projects in either travel or station mode. Twenty-two ECPs were selected as a result, drawn from different professional disciplines including architecture, construction management, engineering, and quantity surveying. All of the interviewees held senior positions in their organisations, ranging from senior professionals to directors. In addition, conforming to the male-dominated nature of the construction industry, all of the interviewees were male. They all had more than 10 years’ experience in the construction sector and at least two years’ experience of working in mainland China. Detailed background information about the interviewees is summarised in Table 1.

Before starting each interview, the interviewer described the purpose of the study, explained the ground rules, and provided assurances about confidentiality. Semi-structured interviews were conducted, in which interviewees were asked prompting questions about their stress (How does stress manifest in you?), consequences of stress (What are the consequences of stress?), coping behaviours (How do you cope with stress?), and motivation in terms of coping behaviours-to-stress expectancy (Based on your experience, how likely is it that the above-mentioned coping behaviours can manage your stress?), stress-to-performance expectancy (Based on your experience, how likely is it that stress will affect your above-mentioned performance?), and valence to performance (How important is performance for you?). On average, all interviews lasted for an hour. The interviews were audio-recorded and contemporaneous notes were taken. To minimize variability of interview practice, all interviews were conducted by the same researcher in the team.

The researcher adopted the techniques of summarising and paraphrasing in order to cross-validate the information collected from the interviewees and minimise the possibility of data distortion. Using the qualitative data collected, a systematic contextual analysis was then conducted.

## 6. Results and Discussion

The interview data were gathered, summarised, and merged into tables using keywords and phrase identification [58,59]. According to the conceptual model developed based on previous related studies (e.g., Leung et al. [18] for stress-performance, Lazarus and Folkman [21] for coping behaviors, and Vroom [28] for motivation), an initial list of keywords was firstly developed under different categories (e.g., problem solving and positive reappraisal for problem-focused coping behaviors; escapism and emotional support for emotion-focused coping behaviors; [21]. Modification was further made within the course of analysis as new categories emerge (e.g., praying for emotional-focused coping). This method has the advantage of supporting the accumulation and comparison of research findings across multiple studies [60]. The qualitative data were then categorised into factors under the conceptual framework (Table 2, Table 3, Table 4 and Table 5). 

### 6.1. Manifestations of Stress

All the interviewees reported that they had experienced stress in their work in mainland China. They identified eighteen symptoms that they encountered when under stress (refer to Table 2). Work stress occurs when there is a discrepancy between an individual’s expected and actual ability to work on his or her work tasks. Interviewees from three of the projects expressed that stress was manifest in terms of difficulty of task and too many tasks to handle. For instance, B-C1 expressed that “…The regulations in mainland China, like construction and E&M regulations, have a great difference from that of Hong Kong. The construction standards in Hong Kong follow the British standard. However, the construction standards in mainland China partly follow the Russian standard and were mainly developed independently. We are not familiar with these local practices. There are many regulations in the mainland practices, like national standards and industrial standards. We are not sure about what regulations to follow in particular situations…”. Meanwhile, C-C1 stated, “I have much work to do … I have unsolved problems (i.e., difficulties) … I am sometimes too busy travelling and working, reversing my living sequence”, which is exactly a demonstration of both qualitative stress (i.e., difficulty of tasks exceeding one’s ability to solve them) and quantitative stress (i.e., large number of tasks exceeding one’s ability to handle them) [61].

Additionally, interviewees expressed that they failed to “let go” of the stressful problems affecting them in their life and work in mainland China. For instance, A-C3 said, “I keep on thinking about problems, like relationship problems with clients and management problems with subordinates, when I am stressed…” Another interviewee, B-A1, even stated, “…I cannot stop myself from thinking…”. Failure to let go of troubles and relax may further cause negative moods, like being irritable, anxious, or even emotionally exhausted. As expressed by A-C2, “I do not want to go to work in the morning when I am too stressed…”, which is a demonstration of emotional exhaustion.

Interviewees also mentioned a number of physical stress symptoms. For instance, they suffered from sleep disorder, when they were under stress. Their poor sleep quality was also claimed to be caused by their failure to let go of the stressors (i.e., the emotional stress symptoms mentioned above). For instance, A-MC1 stated, “I am not able to sleep under stress. I fail to let go of the problems, wake up, and start to brainstorm at midnight…”. In fact, another interviewee, B-C3, mentioned, “I always suffer from headaches. Perhaps it is because I keep on thinking about problems when I am stressed…”, which also demonstrated the impacts of failure to let go of stressful problems on physical stress, such as headache. Furthermore, the interviewees also found themselves suffering from other physical stress symptoms like stomach ache, high heart rate, tiredness, eating disorder, rash, musculoskeletal pain, loss of hair, freezing hands, and frequent toileting. All these affect the ECPs’ daily performance.

### 6.2. Influences of Stress to Task Performance & Early Return Intention

When asked about the consequences of the stress they experienced, the interviewees addressed five performance items, which can be further categorised into two groups: (i) task performance; and (ii) early return intention (refer to Table 3).

#### 6.2.1. Task Performance

It has been known for some time that stress has an impact on the task performance of construction professionals [10] and expatriates [62,63]. The interviewees confirmed that stress had a negative impact on their task performance, such as work ineffectiveness, poor decision-making, poor critical thinking, and frequent mistake making at work. For instance, B-D3 said, “I work slowly and ineffectively when I am under stress because I waste time getting mad about the [poor] working methods of the locals”; C-C1 said, “My mind is less clear when I am under stress … This limits my creativity and my problem-solving ability”; and B-S1 said, “It is easier for me to make mistakes under stress, particularly when my boss urges me to do the work with very limited time. He blames me after finding my mistakes. This, in turn, causes me to experience more stress, in a vicious cycle”. However, some ECPs thought that stress was not necessarily bad for their performance: “(When) I am suffering from stress, I think more effectively. I consider the problem more comprehensively so as to prevent any subsequent problems. Thus, I think stress can help me in solving problems” (B-D1). Also, C-C2 stated, “For me, a certain level of stress is good … I believe that my decision-making can be more straightforward and faster if I am motivated by a certain level of stress. Without stress, I would be stuck in minor issues that slow my decision-making”. Hence, stress can also make a positive contribution to the task performance of ECPs.

#### 6.2.2. Early Return Intention

Moreover, the interviewees said that excessive stress had prompted an intention to return to Hong Kong and end their expatriate assignments. As B-D3 put it, “I was not used to the language, weather, and environment here (i.e., in Beijing, when he first began the assignment). I was very lonely as well, because there were only a few Hong Kong people involved in this project. I needed to face the locals by myself. I worried about my ability all the time. I was too stressed and had thoughts of returning to Hong Kong”. In fact, previous research has confirmed that expatriates under excessive stress have a higher intention to return early to their home country [63,64], which causes substantial financial loss to their organisations. 

### 6.3. Coping Behaviours for Stress

Interviewees were then invited to share how they coped with the stressful work demands (i.e., stressors—which may cause stress if not managed properly); this resulted in 14 items. The items are grouped into eight categories based on the problem- and emotion-focused coping behaviour framework developed by Lazarus and Folkman [21]. The eight types involve planful problem solving and instrumental support seeking—which are problem-focused—and emotional support seeking, escapism, emotional discharge, acceptance, positive thinking, and religious support—which are emotion-focused (refer to Table 4). Consistent with the results of a previous study [20], Chinese expatriates tend to adopt emotion- rather than problem-focused approaches.

#### 6.3.1. Planful Problem Solving

Planful problem solving refers to an individual dealing with a stressor and its influences actively and overtly [65]. Some of the interviewees said that they made a systematic plan to resolve their stressors. For instance, A-D1 stated, “I keep on thinking about and analysing the problems directly … I usually identify the causes first, then list the various possible solutions, and choose the most suitable one”. Some would try to enrich their knowledge in order to solve their problems at work: “In mainland China, the government department can suspend our project simply because of some minor problems. This caused me stress as it affects my project completion… I participate in a course for registered project managers in mainland China. This enriches my ability to do my work in Beijing. I can get recognition from the local staff. This helps me in managing my staff and dealing with external business issues” (B-S2). In fact, planful problem solving is recognised as an effective coping behaviour for both construction professionals and expatriate managers [10].

#### 6.3.2. Instrumental Support Seeking

An individual can also seek instrumental advice and assistance from their colleagues and superiors who may help them to deal with the issues [66]. As B-C2 put it, “I discuss problems actively with my colleagues. For instance, in the case of receiving complaints from clients, I would go to my colleagues directly to find out more about the actual situation and to prevent similar situations from happening again. I would also communicate with the client so as to seek ways to solve the problem. I would also seek support from the Shanghai branch, which has more resources”. Moreover, C-C4 shared that “Due to the cultural differences and pay differential between Hong Kong and Guangzhou staff, conflict occurs between them. Conflicts between local and Hong Kong staff can result in a number of problems, such as team cooperation, obedience of subordinates, and also the creativity and effectiveness of the project team…. I can consult with experienced colleagues and superiors about problems. There are so many staff in my company, there is always someone appropriate for me to consult…”. In addition to consulting local colleagues, some interviewees (e.g., B-D2 and B-D3) also shared their problems with colleagues or friends in Hong Kong to seek their instrumental advice. Consulting experienced people helps ECPs to solve their stressors directly, which certainly helps to relieve their stress. Instrumental support seeking has been found to be effective in mitigating the stress and stressors of expatriates [67].

#### 6.3.3. Emotional Support Seeking

Social networking plays an essential role in expatriates’ adjustment process [68]. Therefore, in addition to instrumental support, ECPs also seek emotional support from their social network in mainland China and/or Hong Kong. Unlike instrumental support seeking, emotional support seeking aims only to release the negative emotions of an individual rather than tackle the problem. In general, interviewees said that they would seek emotional support from not only their colleagues in mainland China, but also families and friends in Hong Kong. For instance, B-S1 mentioned, “My boss sometimes rejects my constructive suggestions, which causes me stress. I will share this with my friends. I call my friends in Hong Kong”. In addition, A-C2 stated, “I am stressed by the separation from my family. For instance, I am not able to nurture my kids closely… I also Skype and QQ [i.e., online communication tools that are popular in mainland China] my wife and family when I am stressed”. Although these families and friends may not help to solve their problems, ECPs found that talking to them helped to release their negative emotions and made them feel recharged. Emotional support seeking has long been recognised as an effective strategy to fight stress and negative emotions [69]. Social and psychosocial supports from host-country mentors and friends and/or colleagues have also been found to be effective in reducing stress for expatriate managers [70]. 

#### 6.3.4. Escapism

In addition to the active behaviours discussed above, some interviewees engaged in passive behaviour to cope with stress. Instead of taking action to solve the problems or the resulting negative emotions, they engaged in escapism, whereby individuals isolate themselves, escape from stress, and/or avoid the problem [71]. Some ECPs said that they would try to put the problem aside temporarily. For instance, B-D2 stated, “I try to put the stress aside, by working on other issues first. I let my stress go over time”. Another interviewee said that he tried to escape from the stressors by avoiding phone calls. “Communication here (Shanghai) sometimes causes me stress… We, as Hong Kong people, are okay at communicating in Mandarin; however, we may not be capable of communicating in Shanghainese. I felt stressed when I failed to understand what they said... I answer fewer phone calls when I am under stress because I want to take a break and leave some personal space for myself to solve the stressful problem first”, stated A-MC2. However, escapism has been found to be a maladaptive coping behaviour in previous studies [72,73].

#### 6.3.5. Emotional Discharge

The interviewees also tried to discharge their negative emotions by engaging in a wide range of activities, of which doing exercise was the most common. The exercises that interviewees mentioned include playing ball games, swimming, hiking, running, and so on. One interviewee said that exercise was the most important way for him to release his stress: “I release my stress by doing exercise, like swimming, running, and hiking. This is one of the most important ways for me to release stress”. (B-A1). In addition to exercise, interviewees also mentioned other activities that they engaged in when under stress, like reading, walking, travelling, listening to music, and sleeping. Other interviewees, A-MC1 and B-S2, referred to drinking and smoking as coping behaviours. For instance, B-S2 said, “I tend to smoke more when I suffer from stress”. Although some interviewees felt that these activities helped to release their stress by making them happier and more relaxed, emotional discharge has been found to be a maladaptive coping strategy for construction professionals [11].

#### 6.3.6. Acceptance

Some interviewees reported that they tried to release their negative emotions by simply accepting the different culture and imperfections of local colleagues. For instance, C-C1 stated, “I need to accept the different work culture and attitudes of the locals. For example, they thought that our comprehensive considerations and reminders were ‘wordy’. I need to acknowledge this difference, so that I would not think that these are confrontational behaviours”. B-D2 also stated, “Working in mainland China, we need to accept the different culture and work practices of the locals. We cannot enforce the Hong Kong way of doing things here. We need to respect the locals’ work practices and try to cooperate with them”. In addition, B-A2 felt that “As an architect, I need to accept that there are imperfections in everything. It is very hard for us to pursue perfection. Everything has both its positive and negative sides. I accept any difficulties or failures, and recognise them as necessary processes of development”. Interviewees thought that accepting and acknowledging their stressors helped to reduce their negative emotions. 

#### 6.3.7. Positive Thinking

Some interviewees expressed that they tend to perceive problems in positive ways, and that this type of positive thinking helped to reduce their stress level. For instance, A-C1 stated, “I would have a positive thought that the problems will be solved someday. I do not need to be too worried about it”. Although positive thinking has no direct contribution to solving the stressors, interviewees expressed that it reduced their negative emotions.

#### 6.3.8. Religious Support

Religious support has been found to be effective in releasing the stress of individuals [74]. This is particularly true for ECPs who need to stay in mainland China alone, being isolated from the social network of their home town. For instance, A-C2 stated, “Because I am a Christian, I pray and can find support from God whenever I face difficulties”. He believed that his religion provided emotional support to him.

### 6.4. The Intimate Relationships between Motivations and Cope Behaviours

In addition to the coping behaviours identified above, the interviewees were asked about their motivations to engage in each of them. Twelve motivational items were collected, which can be categorised into three dimensions using the expectancy theory—including the pathways between coping behaviours and stress expectancy, the pathways between stress and performance expectancy, and the valence of performance (refer to Table 5). According to the coping behaviours, stress, and performance issues mentioned by the interviewees, participants were then asked three questions in the same interview: “Based on your experience, how likely is it that the above-mentioned coping behaviours (refer to Table 4 for the coping behaviors identified by the participants) can manage your stress?”; “Based on your experience, how likely is it that stress will affect your above-mentioned performance (refer to Table 3 for the performance identified by the participants)?”; and “How important is performance for you?”.

#### 6.4.1. Coping Behaviours-to-Stress Expectancy

Problem-focused coping behaviours have long been recognised as effective in managing the stress of individuals [75,76,77]. It is thus reasonable that no interviewee reported low expectancy of the effectiveness of using planful problem solving and instrumental support seeking to reduce their stress. For instance, B-C3 explained why he applied planful problem solving: “I think troubleshooting is very effective in releasing my stress. Besides, it is very important for me to gather much information to back up my briefing to the contractor. I need to be convincing”. However, it was interesting to note that only five interviewees had low expectancy of the effectiveness of emotion-focused coping behaviours—including emotional support seeking, escapism, emotional discharge, and acceptance, all of which are generally recognised as maladaptive in the literature [76]. As B-D3 mentioned, “Playing football is not totally effective. It can only release my stress and keep my mind clear for a short period of time. I will become anxious again after the game”. A-C3 stated, “I would still think of the problem and be stressed again after chatting (with friends)”. The majority of interviewees expressed that they had high expectancy of the effectiveness of emotion-focused coping behaviours, for example B-C3, who discussed the effect of emotional discharge on relieving his stress: “I can be relaxed when I listen to the music that I like ... Listening to the music helps me to put my problems aside and calm down, so as not to let my thoughts become stuck”. Meanwhile, A-MC1 expressed that “Drinking and eating are quite effective in releasing my stress. Drinking can stop my brain from brainstorming, enabling me to sleep”. These, to a certain extent, explain why these ECPs tended to adopt emotion-focused coping.

#### 6.4.2. Stress-to-Performance Expectancy

The link between stress and performance expectancy also determines the motivations of ECPs to cope with their stress. As mentioned in the previous section, the majority of interviewees had high expectancy of the impact of stress on task performance and early return intention. However, some expected the impact of stress on their performance to be low or insignificant. For instance, B-D3 said, “As I have got used to the work culture of the locals, my task performance is less likely to be affected by stress”. Stress will be less likely to affect his task performance after his adjustment to the local work culture. Although some interviewees expressed low expectancy of the impact of stress on task performance, none reported this low expectancy in terms of early return intention. This, to a certain extent, demonstrates the significant impact of stress on the withdrawal behaviours of an expatriate.

#### 6.4.3. Valence of Performance

Previous studies have indicated the role of the perceived importance of expatriate assignment on the effect of stress [78]. The current study found that the valence of performance also contributed to the individual motivations of ECPs to adopt various coping behaviours. All the interviewees who expressed an opinion about this issue reported high valence of their task performance. These interviewees said that task performance was extremely important to them because it contributed to job satisfaction and personal success, and it proved their value to the organisation. As B-D3 put it, “Task performance is important because it proves my value to my company. It gives me a feeling of security”. In the same vein, A-MC3 expressed that “Working in construction projects, we need to make many decisions every day. These decisions have great implications on time and costing of the projects, which are very important. When mistakes happen, we need to fix them immediately. Therefore, this [task performance] is the most important issue”. 

Intention to stay in mainland China, in terms of remaining on the expatriate assignments and completing their job duties, was identified as an important factor for ECPs. “As the market in Hong Kong is saturated, and the market in mainland China is so big, it is important for me to stay in mainland China. Therefore, I need to insist on staying in Beijing” (B-D3). As C-C2 put it, “It is quite important to have the determination to stay [in mainland China]. Indeed, I cherish this [expatriate] opportunity very much. This is because I can perform to my ability in this large market of mainland China. There are many large-scale and special projects here. I am lucky to have amassed certain experience in mainland China, and that I am able to participate in these projects”. The construction industry in mainland China is rapidly expanding and there are comparatively more job opportunities there than in Hong Kong. Hence, five interviewees who expressed an opinion on this said that it had a high valence for them, while no interviewee perceived this as unimportant. 

In general, interviewees placed a high expectancy on the coping behaviours-to-stress relationship and the stress-to-performance relationship; they also expressed a high valence on their performance levels. All these factors contributed to their adoption of various behaviours to cope with their stress in the high-pressure, expatriate environment.

### 6.5. The Resulting Model

**Figure 2:**
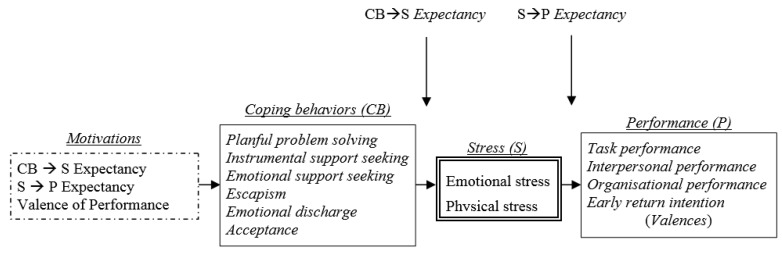
The Resulting Model for Motivations, Coping Behaviours, Stress and Performance of ECPs.

Based on the above qualitative study using data collected from the interviews (refer to Table 2, Table 3, Table 4 and Table 5), the final motivation-coping behaviours-stress-performance model is developed for Hong Kong ECPs in mainland China (refer to Figure 2). 

The model reveals that (i) individuals can cognitively control their own behaviours and decide how much effort to put into coping with their stress under different situations; and (ii) their decisions regarding coping behaviours are determined by the expected effectiveness of the coping behaviours on their stress levels, the expected influence of stress on their performance, and the valence of their performance. Therefore, training on how to cope with stress under different situations is strongly needed for ECPs working in the dynamic, multi-cultural project environment. In order to ensure success in expatriate assignments, it is common for foreign companies to provide training for employees [6,79]. However, traditional training is limited to language, work adjustment, and/or task management. There is a lack of pre-departure training which focuses on ECPs’ stress coping and performance. In fact, amongst the 22 participants in the study, all of them expressed that they did not receive any training for their expatriations, neither pre- nor post-departure training. This also unveils the lack of emphases on the significant impacts of cultural stress on ECPs in our industry.

There is an urgent need for stakeholders to provide pre-departure training to ECPs, helping them to shape their expectancies of various adaptive and maladaptive coping strategies, and their expectancies of the significant influence of stress on performance. For instance, even though previous survey study indicates that task performance of ECPs is significantly affected by their physical and psychological health [80], the results of the in-depth interviews reveal that some of the ECPs still consider those associations weak. On the other hand, even though emotional-focused coping behaviours, such as escapism, have been found to be maladaptive in previous studies [80], the study also revealed that some participants still see it as a useful mean to cope with their stress. 

In fact, shaping individuals’ perceptions is an anchor for long-term change in behaviours [81]. Therefore, instead of simply introducing different types of stress-coping strategies, the different manifestations of stress and its associations with different motivational elements (i.e., coping behaviours-to-stress expectancy, stress-to-performance expectancy, and valence of performance) in the stress management process should be taken into account when designing the training for expatriate professionals. 

In addition, the study also reveals that ECPs are aware of the impact of work stress, in terms of demand and ability balance, however, not enough attention is being paid to their emotional and physical states. On the other hand, the majority of existing stress management training tend to focus on the introduction of stress management skills or practices (e.g., time management or problem solving skills). There is a lack of emphasis on the personal awareness of stress at affective (emotional stress) and physical (physical stress) levels. Hence, in addition to training, regular monitoring of ECPs’ emotional and physical stress levels is also necessary to ensure that they are working at the optimized stress level. This can be done via regular online survey measurements and/or real-time monitoring of bioindicators of stress, such as body temperature and blood pressure. 

## 7. Limitations and Further Research

To explore the firsthand experience of motivations, stress, coping strategies, and performance of ECPs, qualitative interviews were conducted with ECPs working in three multi-cultural projects in Mainland China. A total of 22 ECPs participated in the current study. This relatively small sample size may affect the representativeness of the study results. However, a large sample size is not necessarily beneficial, since it does not facilitate the depth of the data to be collected [82]. In addition, the validity and reliability of the study results were ensured by a number of credible data collection and analysis procedures, such as the following: (i) the purposive sampling method was described explicitly and it was ensured that all participants were qualified Hong Kong construction professionals who had direct expatriate experience of working in Mainland China; and (ii) multiple sources of evidence were used, including audio-recordings and on-the-spot note-taking, to ensure the reliability of the data [83]. 

While qualitative methodology does not pursue generalizability due to the ontological and epistemological stands of the study, transferability can be achieved based on the depth and vividness of data and result descriptions (e.g., [84,85]). Since the enforcement of the open door policy, China has attracted many international professionals. These international expatriates, with their different cultural values, may encounter even more difficulties in managing their stress on the Mainland than Hong Kong ECPs. In addition, despite China’s swift development, the rapid growth of various cities worldwide, such as in India and the U.S.A., has also increased demands on construction professionals from all over the world. The results of the current study not only provide recommendations for Hong Kong ECPs who have to work in Mainland China, but also provide information with which expatriates can better understand the Mainland context and can better choose appropriate coping strategies in an expatriate environment. 

The results of the current qualitative study do not only cross-validate the results of the previous quantitative studies on the related topics (e.g., [80]), but also provide insights on how and why stress and performance of ECPs are affected by different motivational factors and coping behaviors. Based on the study results, intervention studies are recommended to investigate the effectiveness and efficiency of various stress management training for ECPs at pre-departure and post-departure stages. A longitudinal approach would be need to keep track of the effects of different training contents and activities on stress and performance of ECPs.

## 8. Conclusions

To investigate the role of motivation in the stress management process, 22 in-depth interviews were conducted using Hong Kong ECPs in mainland China as a sample. In addition to the three types of stress (emotional stress, and physical stress), the study identified three main constructs for motivation (coping behaviours-to-stress expectancy, stress-to-performance expectancy, and valence of performance), five coping behaviours (planful problem solving, instrumental support seeking, emotional support seeking, escapism, and emotional discharge), and two types of performance (task performance and early return intention). The results confirm that motivation governs ECPs’ coping behaviour adoptions, which further affect ECPs’ stress and performance levels. Stakeholders are recommended to review, and revise if needed, the objectives and contents of existing pre-departure training, so as to include key elements such as personal awareness of stress at different levels (cognitive, affective, and physical), expectancies of various adaptive and maladaptive coping strategies (e.g., escapism as a maladaptive coping strategy) on stress, and expectancies of the influence of different types of stress on performance. These results form a basis for further longitudinal studies on stress management interventions for ECPs.

## Figures and Tables

**Table 1:** Background Information of Interviewees.

	Characteristic	Position	Experience in China (years)	Station/Travel
Participants				
**Project A (Shanghai)**				
A-D1		Assistant Manager	3	Travel
A-MC1		Senior Manager	10	Station
A-MC2		Project Manager	10	Station
A-MC3		Engineer	10	Station
A-C1		Executive Engineer	4	Station
A-C2		Director	3.5	Station
A-C3		Senior Building Services Engineer	4	Station
A-C4		Assistant Director	15	Travel
**Project B (Beijing)**				
B-D1		Project Manager	4.5	Station
B-D2		Senior Project Manager	3.5	Travel
B-D3		Assistant Construction Manager	4.5	Station
B-A1		Architect	4	Station
B-A2		Associate Architect	4	Station
B-S1		Senior Project Manager	4	Station
B-S2		Project Manager	2	Station
B-C1		Engineer	3	Travel
B-C2		Director	8	Travel
B-C3		Registered Engineer	10	Travel
**Project C (Guangzhou)**				
C-C1		Associate Director	5	Station
C-C2		Associate Director	17.5	Travel
C-C3		Director	16	Travel
C-C4		Director	10	Travel

Note: The first digit of the participant code refers to the project to which he belonged (i.e., A–C). The second digit refers to the type of company to which he belonged, in which D = developer, MC = main contractor, C = consultant, A = architect firm, and S = sub-contractor.

**Table 2:** Summary of Stress Symptoms Identified by Interviewees.

	Participants	Project A (Shanghai)	Project B (Beijing)	Project C (Guangzhou)
Stress				
**Work stress**				
1. Difficult tasks (exceeding personal ability)		MC1, MC3, C3, C4	D1, D2, C1, A1, A2	C1, C3, C4
2. Too many tasks (exceeding personal ability)		MC3	D1	C1
**Emotional stress**				
3. Failure to let go		D1, MC2, C2, C3, C4	D2, A1, A2, C3	C2
4. Anxious		D1	S1, C1	C2, C3
5. Irritable		MC1, C2, C3	D1, D2, D3, S2, C1, C3	C2, C3, C4
6. Emotional exhaustion		C2	-	-
7. Negative mood		-	-	C4
**Physical stress**				
8. Sleep disorder		D1, MC1, C2, C3, C4	S1, S2, A1, C1, C2, C3	C1, C2, C3, C4
9. Stomach ache		MC1	D2, A1	C4
10. High heart rate		MC1	D1, D2	-
11. Tiredness		-	A1	C1
12. Headaches		-	S2, C3	-
13. Eating disorder		C2, C4	-	-
14. Rash		-	-	C1
15. Musculoskeletal pain		C3	-	-
16. Loss of hair		MC1	-	-
17. Freezing hands		C1	-	-
18. Frequent toileting		C1	-	-

Note: Refer to Column 1 of Table 1 for the coding of interviewees in the three projects (second digit).

**Table 3:** Summary of the Consequences of Stress as Identified by Interviewees.

Consequences of Stress	Sample Scripts	Interviews		
		Project A (Shanghai)	Project B (Beijing)	Project C (Guangzhou)
Stress → Task Performances				
1. Work effectiveness	B-C1: I **work** slowly and **ineffectively** when I am under stress because I waste time getting mad about the (poor) working methods of the locals. However, I am able to understand their culture and know how to react to their different work culture now. A-MC2: When I encounter stress, I hope to solve the work problem earlier. This can result in **higher effectiveness**. A-C4: I **work** much **slower** under stress. Also, I am distracted and less able to concentrate at work.	MC2(+), C3(−), C4(−)	D1(+), D2(−), C1(−), A1(−), C3(−)	-
2. Decision-making	B-C1: Suffering from stress, I become irritable, which causes me to **make decisions** very quickly, without thorough consideration. The decisions I make under stress may not be the most comprehensive. C-C2: For me, a certain level of stress is good. It keeps me moving forward. Perhaps my stress level is not very high at the moment. I believe that my **decision-making** can be more straightforward and faster if I am motivated by a certain level of stress. Without stress, I would be stuck in minor issues that slow my decision-making.	-	C1(−)	C2(+)
3. Poor critical thinking	A-C2: I cannot **think** of the problem **critically** and analyse it when I suffer from stress. C-C1: My **mind** is less clear when I am under stress. I may have confrontations with counterparts or other related parties. This limits my creativity and my problem-solving ability.	C2	-	C1
4. Mistakes	B-S1: It is easier for me to make **mistakes** under stress, particularly when my boss urges me to do the work with very limited time. He blames me after finding my mistakes. This, in turn, causes me to experience more stress, in a vicious cycle. B-S2: My memory is poorer when I am under stress, as my mind focuses on the troubles, and forgets the other issues. As I concentrate on the troubles, I may make **mistakes** or even ignore other issues. A-C1: Under a heavy workload, I need to take on more tasks in a tighter time frame. This causes me to make **mistakes** more easily and frequently.	MC2, MC3, C1	S1, S2,	-

Stress → Task Performances				
5. Early return intention	B-D3: At the beginning, when I first came to BJ, I was not used to the language, the weather, and the environment here. I was very lonely as well, because there were only a few Hong Kong people involved in this project. I needed to face the locals by myself. I worried about my ability all the time. I was too stressed and had thoughts of **returning to HK**. A-C2: I have thought of ending my work in SH and **relocating back to HK**. I have doubted whether I am suitable to work in SH. A-C3: I have thought of **early return to HK**. This is because I hope I can spend more time with my family. C-C2: As my kids are still young and I am tired of travelling, I hope to have more time to **station in HK**. Therefore, 50% of my thoughts are wanting to go back to HK. This is also the reason I relocated from BJ and SH to GZ and SZ now. However, if I station in HK for a long time, I may be bored and want to travel again.	C2, C3	D3, S2	C2

Note: Refer to Column 1 of Table 1 for the coding of interviewees in the three projects (second digit). (+)/(−) refer to positive/negative influences of work stress (if there were opposite views raised by interviewees).

**Table 4:** Summary of Coping Behaviours Identified by Interviewees.

Coping Behaviours → Stress	Sample Scripts *(To relieve stress, …)*		Interviews	
		Project A (Shanghai)	Project B (Beijing)	Project C (Guangzhou)
Problem-Focused Coping Behaviours				
Planful problem solving				
1. Planning of problem solving	C-C3: I **plan** how to fix the problem that causes me stress. For instance, as the project duration is very tight, we need to achieve this target through better (project) **planning**. Planning can also help relieve our stress.	D1, MC1, MC2, C4	S2, C3	C3, C4
			
**Instrumental support seeking**				
2. Consulting colleagues	B-C2: I discuss problems actively with my **colleagues**. For instance, in the case of receiving complaints from clients, I would go to my colleagues directly to find out more about the actual situation and to prevent similar situations from happening again. I would also communicate with the client so as to seek ways to solve the problem. I would also seek support from the SH branch, which has more resources. C-C4: I can consult with **experienced colleagues and superiors** about problems. There are so many staff in my company, there is always someone appropriate for me to consult.	MC3, C1, C2	D2, D3, C2	C1, C2, C3, C4
		
**Emotion-Focused Coping Behaviours**				
**Emotional support seeking**				
3. Seeking emotional support from friends	B-S1: My boss sometimes rejects my constructive suggestions, which causes me stress. I will share this with my **friends**. I call my friends in HK and discuss the problem.	MC2, C1, C2, C3	D1, S1, S2, A1, A2, C2	C3, C4
4. Seeking emotional support from colleagues	C-C4: I live with one of my **colleagues** now. He is my old university classmate and is currently working at the Fushan office. We pour out our troubles to each other.	MC2	D3	C3, C4
5. Seeking emotional support from family	A-C2: …I also Skype (i.e., an online communication tools used worldwide, but blocked in mainland China) and QQ (i.e., an online communication tool used in mainland China) my **wife and family** when I am stressed.	C2, C4	-	C2

Escapism				
6. Putting the problem aside (temporarily)	B-D2: I try to **put the stress (stressor) aside**, by working on other issues first. I let my stress go over time.	-	D1, D2	-
7. Avoiding phone calls	A-MC2: I answer **fewer phone calls** when I am under stress because I want to take a break and leave some personal space for myself to solve the stressful problem first.	MC2	-	-
**Emotional discharge**				
8. Exercise	B-A1: I release my stress by **doing exercise**, like swimming, running, and hiking. This is one of the most important ways for me to release stress. Doing exercise is the happiest event for me. Therefore, I will go to exercise whenever I am stressed. This is healthy as well. Happiness is closely linked with health. I only choose healthy ways to release my stress, so as to make myself happy.	MC3, C2	D3, S1, S2, A1, C1, C2	C1, C3
9. Reading/watching films/watching TV	C-C4: This habit (**watching films**) is very important for me. This can occupy my brain and help me stop thinking about the work issues after working hours. I can then go back to the problem calmly after my negative emotions have faded with time.	MC2, C4	S2, A2	C3, C4
10. Walking around/sightseeing	B-A2: I like **exploring and experiencing the different local culture** in BJ. I like seeing how people live here. My stress can be released by exploration of these different cultures.	-	S1, A2	-
11. Travelling	A-MC1: It is important for me to have a break and **leave the mainland environment** after a certain period of work, let’s say for half a year. For example, I will go back to HK or to Australia.	MC1	D3, S2	C2
12. Listening to music	B-C3: I listen to some **HK music** when I am stressed in BJ. I have more than 500 HK songs.	-	S1, C2, C3	C1
13. Sleeping	B-S1: **Sleeping** is the most effective. This is because I can get myself refreshed both physically and mentally.	MC2, C1, C3	D3, S1, A1	-
14. Drinking and eating	A-MC1: I don’t like hanging around. I like going home after work, **drinking wine**, which is usually accompanied by **food**. Therefore, I am becoming fatter. However, I have a more regular lifestyle (i.e., no drinking wine and eating extra food) when I am not under stress.	MC1	-	-
15. Smoking	B-S2: I tend to **smoke** more when I suffer from stress.	-	S2	-

Acceptance				
16. Accepting the different culture	B-D2: Working in mainland China, we need to **accept** the **different culture** and work practices of the locals. We cannot enforce the HK way of doing things here. We need to respect the locals’ work practices and try to cooperate with them.	-	D2	C1
17. Accepting the imperfections	B-A2: As an architect, I need to **accept** that there are **imperfections** in everything. It is very hard for us to pursue perfection. Everything has both its positive and negative sides. I accept any difficulties or failures, and recognise them as necessary processes of development.	-	A2	C4
**Positive thinking**				
18. Reappraisal of the problem positively	A-C1: I would have a **positive thought** that the problems will be solved someday. I do not need to be too worried about it.	C1	-	C1, C4
**Religious support**				
19. Praying	A-C2: Because I am a Christian, I **pray** and can find support from God whenever I face difficulties.	C2	-	-

Note: Refer to Column 1 of Table 1 for the coding of interviewees in the three projects (second digit).

**Table 5:** Summary of Motivations Identified by Interviewees.

	Participants	Sample scripts	High			Low		
Motivations			*Project A*	*Project B*	*Project C*	*Proj A*	*Proj B*	*Proj C*
Coping Behaviours → Stress *Expectancy*								
Problem-focused coping								
1. Planful problem solving		C-C3: If the stress is caused by a certain problem, **making plans** to solve the problem would be the **most effective** method to release the stress.	D1, MC2, C1, C4	C3	C3	-	-	-
2. Instrumental support seeking		C-C2: **Sharing the problem** with my **colleagues** is more **important**, as it increases the chance of solving the problem	MC3	D3	C1, C2, C3, C4	-	-	-
Emotion-focused coping								
3. Emotional support seeking		A-MC2: Through the process of **discussion** (with friends or colleagues), no matter whether the discussion is focused on the stressful problem or not, I can brainstorm and find out the problem naturally. This can directly **release my stress**.	MC2, C1, C2, C3, C4	D1, D3, S1, S2, A1, A2	C1, C2, C4	-	-	-
4. Escapism		A-C3: By **staying alone** in a quiet environment, I can have a clear mind and then think of the solution to the problem later. B-D1: It (putting the problem aside) is **not extremely effective**, because I still need to solve the problem later on.	MC2, C3	D2	-	-	D1	-

5. Emotional discharge	B-S1: **Swimming** is quite **effective** in releasing my stress, as it can clear my mind.B-D3: **Playing football** is **not totally effective**. It can only release my stress and keep my mind clear for a short period of time. I will become anxious again after the game.	MC1, MC2, MC3, C1, C2, C3, C4	S1, S2, C1, A1, A2, C3	C1, C4	MC2		D3	C3, C4
6. Acceptance	C-C1: Because cultural difference is the major source of stress in the mainland environment, it is very important to **acknowledge** it. It can help to prevent interpersonal conflict and find solutions to problems. This deals with the **roots of expatriate stress** in the mainland.	-	A2	C1, C4	-	-	-
7. Positive thinking	A-C1: **Thinking positively** can help **release** my stress. This is because it is impossible for us to worry about all the problems.	C1	-	C1, C4	-	-	-
8. Religious support	A-C2: **Praying** is an **extremely effective** way for me to cope with my stress.	C2	-	-	-	-	-
**Stress → Performance *Expectancy***								
9. Task performance	A-C1: Stress is **quite likely** to affect my **task performance** by causing me to make mistakes more easily.B-D3: Because I have gotten used to the working culture of the locals, my **task performance** is **not** that likely to be affected by stress.	MC2, MC3, C1, C2, C3	D1, S1, S2,	C1, C4	-	D3, C1, A2	-
10. Early return intention	A-C2: Both work and expatriate stresses **cause** me to have thoughts of **early return** to HK.	C2, C3	D3, S2	-	-	-	-
***Valence* of Performance**								
11. Task performance	B-D3: **Task performance** is **important**. This is because it proves my value to my company. It gives me the feeling of security.	MC2, MC3, C1, C2, C3, C4	D1, D3, S1, S2, C1, A1, A2, C3	C1, C2, C4	-	-	-
12. Intention to stay	C-C2: It is quite **important** to have the **determination to stay** in mainland China. Indeed, I cherish this opportunity very much. This is because I can perform to my ability in this large market in mainland China. There are many large-scale and special projects in here. I am lucky to have amassed certain experience in mainland China, and that I am able to participate in these projects.	C2, C3	D3, S2	C2	-	-	-

Note: Refer to Column 1 of Table 1 for the coding of interviewees in the three projects (second digit). Only expectancy(ies) and valence(s) regarded as motivations to cope are included in this table.
